# Static magnetic field assisted thawing improves cryopreservation of mouse whole ovaries

**DOI:** 10.1002/btm2.10613

**Published:** 2023-10-18

**Authors:** Liyuan Zhang, Mengqiao Chi, Yue Cheng, Zhongrong Chen, Yunxia Cao, Gang Zhao

**Affiliations:** ^1^ School of Basic Medicine Anhui Medical University Hefei China; ^2^ School of Biomedical Engineering Anhui Medical University Hefei China; ^3^ Department of Obstetrics and Gynecology Reproductive Medicine Center, The First Affiliated Hospital of Anhui Medical University Hefei China; ^4^ NHC Key Laboratory of Study on Abnormal Gametes and Reproductive Tract (Anhui Medical University) Hefei China; ^5^ Department of Electronic Engineering and Information Science University of Science and Technology of China Hefei China

**Keywords:** fertility cryopreservation, magnetic induction intensity, ovarian tissue, oxidative stress, transplantation

## Abstract

Ovarian tissue cryopreservation is considered to be the only means to preserve fertility for prepubertal girls and women whose cancer treatment cannot be postponed. However, ovarian tissues are inevitably damaged by oxidative stress during cryopreservation, which threatens follicle survival and development, and thus affects female fertility. Therefore, reducing tissue oxidative stress injury is one of the major challenges to achieving efficient cryopreservation of ovarian tissues, especially for whole ovaries. Here, we proposed a new method to improve the antioxidant capacity of whole ovaries during cryopreservation, static magnetic field assisted thawing. The results demonstrated that the antioxidant capacity of the ovarian tissue was significantly improved by static magnetic field treatment. In addition, ovarian tissue allograft transplantation was carried out, which successfully achieved vascular regeneration and maintained follicular development. The findings of this study not only provide a new reference for the preservation of female fertility, but also is a major step forward in the cryopreservation of tissues and organs. It will have good application prospects in the field of assisted reproduction and cryo‐biomedicine.

**Translational Impact Statement**

Our study introduces a new method, static magnetic field‐assisted thawing, to enhance the antioxidant capacity of whole ovarian tissue during cryopreservation. Our method enables advances in ovarian tissue cryopreservation. The experimental results prove its clinical potentials, which is not only conducive to the preservation of female fertility, but also opens up new ways for tissue and organ cryopreservation.

## 1 INTRODUCTION

With breakthroughs in oncology diagnosis and therapies, the detection rate of all kinds of malignant illnesses and patient survival rates have grown considerably.^1^, ^2^, ^3^, ^4^, ^5^ Nevertheless, cancer treatments has been identified as a major factor contributing to reduced female fertility, greatly impacting the quality of life of female survivors.^6^, ^7^, ^8^ The advancement of novel tissue cryopreservation techniques has provided hope and insight into the field of female fertility preservation. Ovarian tissue (OT) cryopreservation is a promising technique for safeguarding and preserving the fertility of young women.^9^, ^10^, ^11^, ^12^ In addition, OT cryopreservation is most suitable for pre‐pubescent girls with cancer and women of childbearing age for whom delayed radiotherapy is not advisable. It allows OT to be frozen immediately after cancer diagnosis without delaying treatment of the disease.^13^, ^14^, ^15^, ^16^ Therefore, OT cryopreservation is of great significance. And it has also been proved that is an effective method of fertility preservation by a number of authorities, including the American Society for Reproductive Medicine.^17^ OT cryopreservation has developed rapidly in recent years and currently, over 200 babies have been born worldwide using this technique.

Despite the continuous development of OT cryopreservation techniques, it has been observed that tissue damage remains a constant and inevitable factor due to the heterogeneous cellular composition of ovaries.^18^ Cryopreserved tissues are sensitive to oxidative stress and apoptosis, resulting in follicular apoptosis and stromal cell death.^19^, ^20^, ^21^ This excessive reactive oxygen species (ROS) production has negative effects on reproduction, including reduced development of embryos and the emergence of progeny diseases, despite the fact that ROS are naturally produced during normal intracellular physiology.^22^, ^23^, ^24^, ^25^ It is thus essential to identify and refine techniques to enhance the oxidative scavenging potential of tissue. Fertility preservation centers globally are refining techniques to enhance oxidative scavenging potential, including improving cryopreservation protocols, applying antioxidants, and utilizing nanotechnology, among other approaches.^7^, ^26^, ^27^ Evidence suggests that various commonly used cryoprotectants (CPAs), including glycerol, sucrose, and dimethyl sulfoxide (DMSO), are effective ·OH scavengers.^28^ Additionally, synthetic and plant‐derived antioxidant substances, such as melatonin, resveratrol, and aloe vera extract, have been tested in OT cryopreservation.^29^, ^30^ Additionally, vitrification of OT using a metal containment system and found no changes in antioxidant parameters or viability of the rewarmed tissue samples.^31^

The effectiveness of static magnetic field (SMF) in biological systems has long been proved.^32^, ^33^ SMF can penetrate biological tissues and interact directly with mobile charges, such as proteins and ions, as well as magnetic compounds within these systems.^34^ Moreover, it can potentially reduce oxidative stress, gene mutation, and apoptosis.^32^ According to recent studies, researchers have sought to investigate the effects of SMF on the antioxidant defense systems of animal and plant cells, and it has been found that SMF have antioxidant properties. Glinka et al. observed that an SMF generated by a permanent magnet may present slight antioxidant activity, while other reports have indicated that SMFs can reduce fluoride ion‐induced oxidative stress and normalize antioxidant enzyme activity.^35^, ^36^, ^37^ Recent studies have further demonstrated that SMF have antioxidant properties and can change the redox balance of mouse fibroblasts in vitro, as well as increasing the resilience of oocytes to damage during cryopreservation.^36^, ^38^ Despite all this, there is currently little evidence to suggest that SMFs can reduce cryo‐damage or inhibit the formation of ROS in cryopreserved ovarian tissue (OT).

**SCHEME 1:**
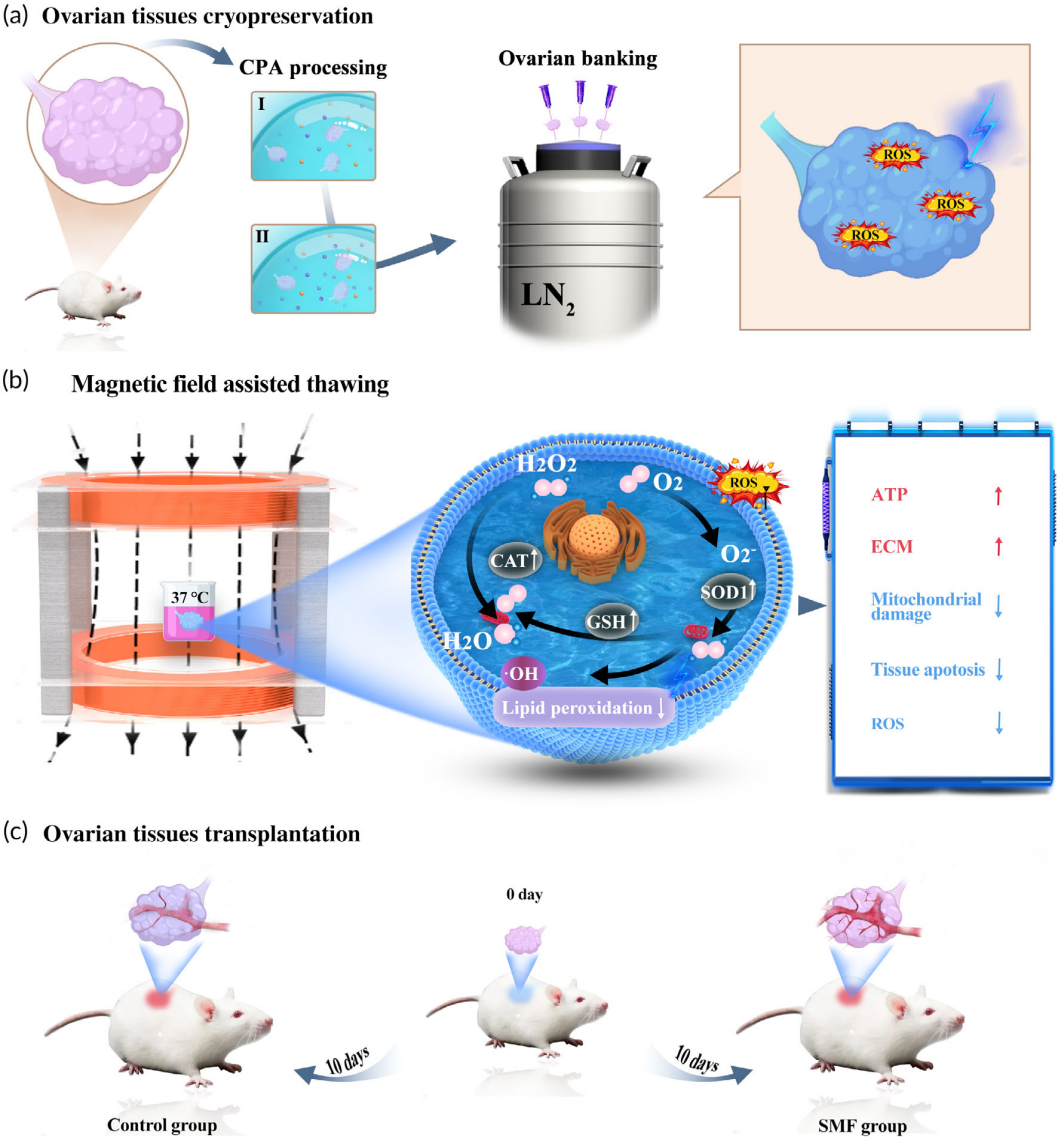
Schematic illustration of the OT cryopreservation, magnetic field‐assisted thawing and transplantation. (a) Acquisition, CPA addition, cooling and storage of OT. (b) SMF‐assisted OT thawing and changes in the content of antioxidative substances in tissue. (c) Tissues transplantation.

This research aims to investigate protective effects of SMF on cryopreserved whole OT, particularly in regard to their antioxidant properties in biological tissues. In this study, we presented a novel SMF assisted thawing method to enhance the antioxidant capacity of cryopreserved whole OT (Scheme 1). The SMF generation device consisted of two Helmholtz coils, and the magnetic induction intensity was regulated by adjusting the current magnitude. It only requires the OT to be placed in the coil during thawing. This approach is advantageous in that it was straightforward to use and could be applied in various scenarios. To our knowledge, the effects of SMF on the thawing phase have not been investigated, so we first assessed how different magnetic induction intensities affect OTs. We further investigated the effects of magnetic fields on cryopreserved OT, along with possible underlying mechanisms. Our results revealed that samples treated with SMFs had increased follicular structural integrity, with improved proliferative capacity. Additionally, the thawed OTs were transplanted to assess their quality and function after cryopreservation. All these proved the feasibility of SMF assisted thawing in clinical OT cryopreservation. Furthermore, the appropriate intensity of SMF was found to increase the total superoxide dismutase (T‐SOD) contents. In short, this cost‐effective and easy‐to‐operate method provides a new reference for the preservation of female fertility in the field of assisted reproduction, and it is also an important attempt of organ cryopreservation.

## 2 RESULTS

### 2.1 Simulation and characterization of static magnetic field

**FIGURE 1:**
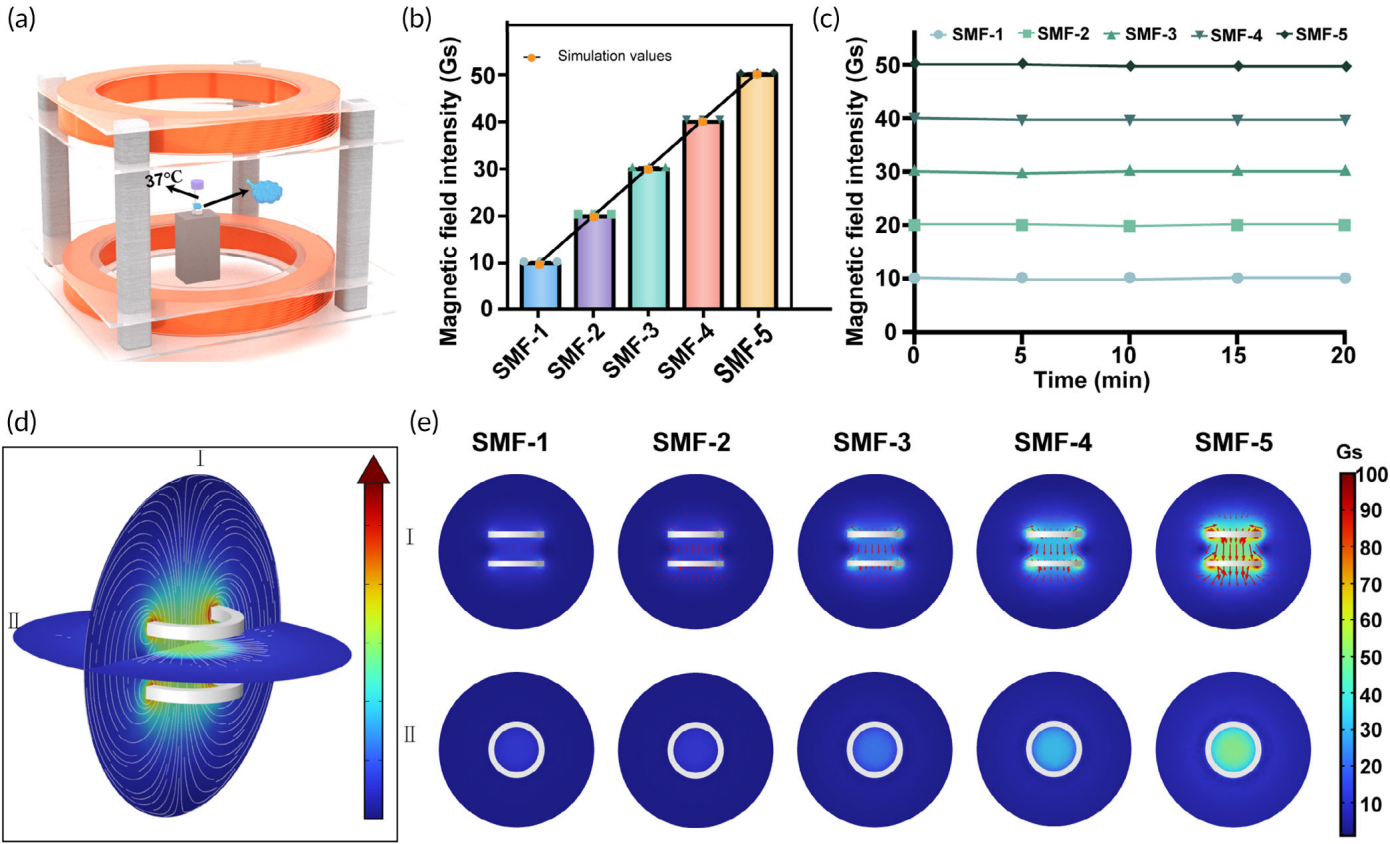
Experimental apparatus for SMFs, with its simulation and characterization. (a) Diagrams of the self‐designed magnetic apparatus. (b) Magnetic induction intensity values and simulated values (*n* = 3). (c) The magnetic induction intensity value at the center of the OT. (d) The general law of the magnetic induction direction distribution in the coil. (e) Simulation of SMF on cross section I and II (Gs: Gauss, a unit of magnetic field strength that is commonly used in the field of magnetism).

The SMF generator has been successfully prepared and characterized, as shown in Figure 1. The magnetic induction intensity applied to each group are analyzed to assess the steady state of the SMF. The OT was positioned geometrically centered in the coil, as depicted in Figure 1a. To determine the magnetic induction intensity, simulations are also conducted. Figure 1b represents the measured and simulated magnetic induction intensity values for different groups, indicating a correspondence between the simulated and measured values. In addition, the magnetic induction intensity within the Helmholtz coil remained constant throughout the experiment, as evidenced by Figure 1c demonstrating the stability of the generated magnetic field by the coil. To verify the magnetic induction intensity at the tissue location, simulations are performed on two cross sections (I and II), as in Figure 1d. The simulation results for section I demonstrate a consistent magnetic field direction from top to bottom with no significant directional deviation, indicating a stable magnetic field. This is further supported by the simulation results of cross‐section II, showing that the OT is in a uniform magnetic field (Figure 1e). Importantly, there was no significant increase in temperature within the magnetic field coil space during the experiment, indicating that the effect of the magneto‐generated heat reaction on the experimental study can be neglected (Figure S1).

### 2.2 Analysis of morphology and extracellular matrix

**FIGURE 2:**
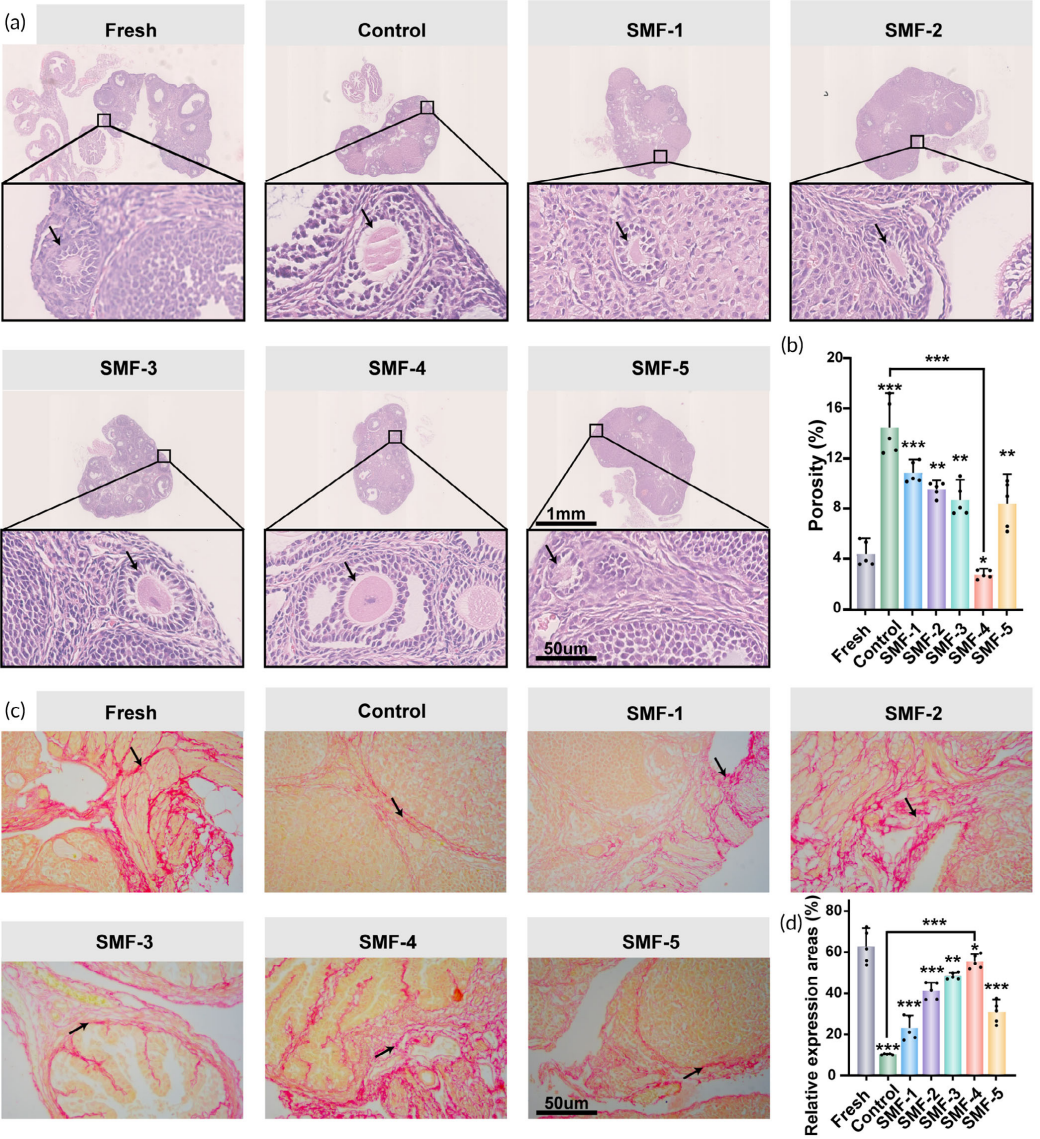
Effect of follicle morphology and collagen fibers in cryopreserved OTs. (a) Structure of OT for each group, scale bar: 50 μm. (b) The ratio of the interstitial stromal area in each OT (*n* = 5, data presented as mean ± SD). (c) Staining of matrix collagen fibers, scale bar: 50 μm. (d) The corresponding quantification of Sirius red dyeing (*n* = 5, data presented as mean ± SD).

Various cells play a crucial role in the female reproductive process and fertility. Among them, follicles and the integrity of oocytes are particularly important for OT function and gamete production. To assess the effect of SMFs on cryopreserved OTs and related indicators, morphological analysis experiments were conducted, as indicated in Table S1. Figure 2a provides an overview of the OTs from different groups and representative images of follicles. The fresh group exhibited significantly better follicle morphology compared to the cryopreservation group. The SMF‐treated groups show better follicle morphology compared to the control group (without SMF treatment). However, it is worth noting that some samples exhibited areas of decreased stromal density or irregular follicle and oocyte morphology. The SMF‐5 group showed a deterioration in follicular morphology compared to SMF‐4, suggesting a potential counter‐effect of the SMF. The medulla, although relatively small in size, plays a crucial role in the ovarian tissue. While it may not contain follicles internally, it houses a dense network of blood vessels. These blood vessels are responsible for providing nourishment to the surrounding follicles, ensuring they receive an adequate supply of oxygen and nutrients. Additionally, the medulla helps in clearing metabolic waste products generated by the follicles, maintaining a healthy and functional ovarian environment. Furthermore, the porosity of the OT medulla was calculated to further investigate the impact of SMF on sample morphology. Figure 2b shows that the medulla porosity gradually decreased with increasing magnetic induction intensity, with the control group exhibiting the highest porosity and SMF‐4 group showing the lowest. In each assay, the SMF‐4 group also showed less porosity than the SMF‐5 group.

Extracellular matrix (ECM) plays a crucial role in determining the quality of cryopreserved OT. In order to examine the distribution of collagen fibers in the ECM, tissue sections were stained with Sirius red. Representative sections stained with Sirius red are shown in Figure 2c. Sirius red staining is decreased in the cryopreserved samples than in the fresh group (Figure 2d). However, the samples treated with SMF (SMF‐1 to SMF‐4) exhibited a gradual increase in collagen fibril content compared to the control group. It decreased in the SMF‐5 group. These findings suggest that SMF had a significant impact on the collagen fiber content of cryopreserved OT, which is consistent with the results obtained from the HE staining. Figure S2a, b represents the stress–strain curves of the tissue subjected to different MF intensities during thawing. The figures illustrate the relationship between engineering stress and engineering strain. It is evident that mouse ovarian tissue behaves as a nonlinear material, and under the same engineering strain, the engineering stress increases with an increase in magnetic induction intensity. For example, when the engineering strain of the tissue is 0.3, the engineering stress is 0.031 MPa in the control group, 0.041 MPa in the SMF‐2 group, and 0.070 MPa in the SMF‐4 group. This analysis indicates that the mechanical strength of the tissue significantly increases with the increase in magnetic induction intensity.

### 2.3 Ultrastructure analysis

**FIGURE 3:**
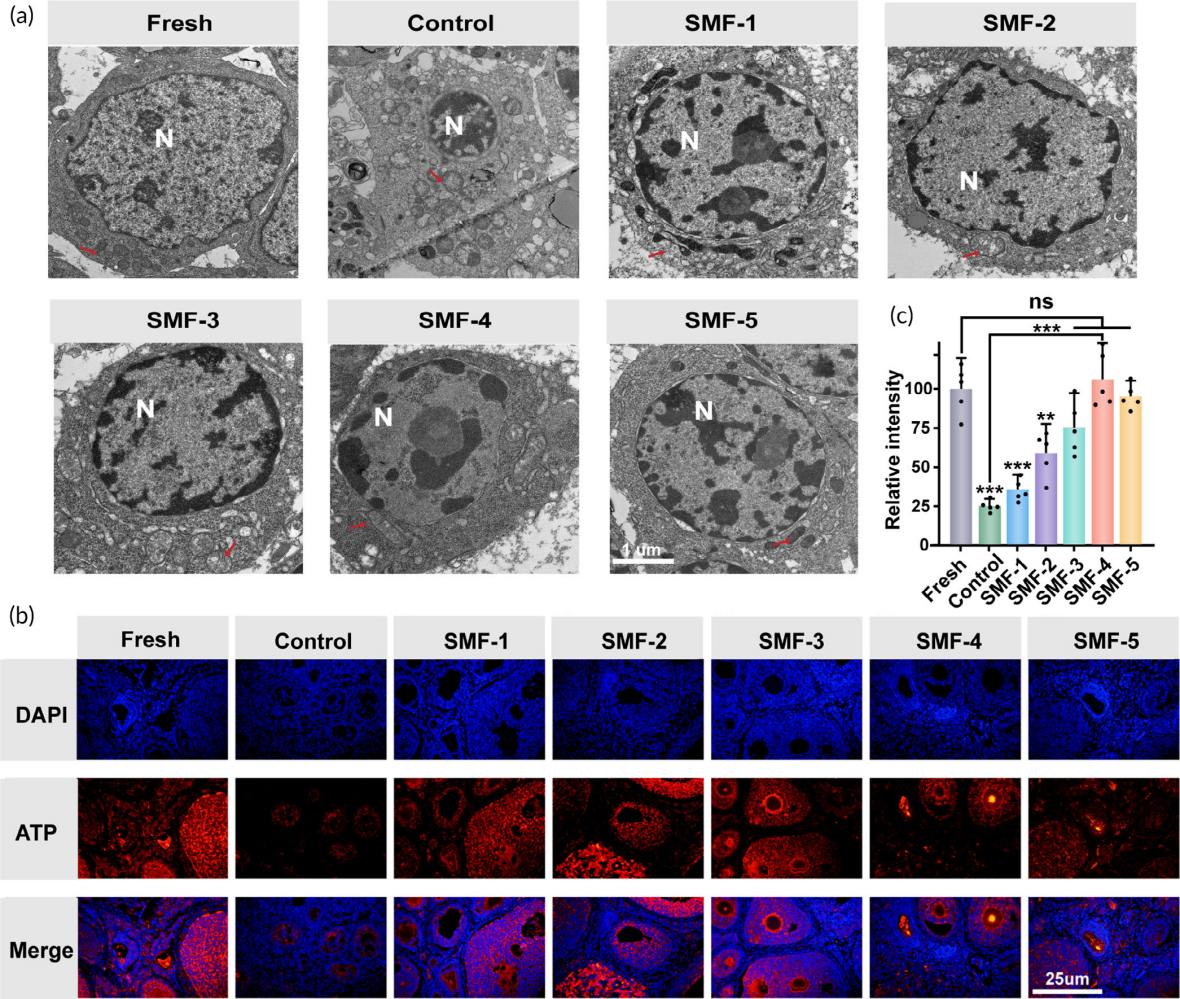
Ultrastructural analysis of granulosa cells with different thawing treatments. (a) Transmission electron microscope images, scale bar: 1 μm. (b) Immunofluorescence staining for ATP (red), and nucleic (blue), scale bar: 25 μm. (c) The corresponding quantification of ATP (*n* = 5, data presented as mean ± SD).

The quality of cryopreserved OT was further assessed using transmission electron microscopy (TEM) to examine the structure of oocytes and granulosa cells (GCs). The results obtained from the morphological analysis of tissue thawed under SMF exposure are presented in Figures 3 and S5. In the control group, TEM observations showed the separation of the zona pellucida from the oocyte membrane and an enlarged perivitelline space. However, the oocytes treated with SMF exhibited a normal perivitelline space surrounding the oocyte and a clear and compact connection structure with the GCs (Figure S2c). Figure 3a illustrates the ultrastructural morphology of granulosa cells in the fresh group, characterized by smooth cell membrane surfaces and normal mitochondrial morphology. After cryopreservation, an increase in the number of vacuoles in the cytoplasm of cells was observed. However, the cells subjected to SMF exposure were protected from cryo‐damage and exhibited reduced cytoplasmic damage characteristics. Moreover, cryopreservation may disrupt the structure and membrane of mitochondria, but the mitochondrial morphology of cells subjected to SMF treatment remained normal, especially in the SMF‐4 group. Additionally, ATP staining and quantitative analysis were performed, and the SMF‐4 group showed higher relative fluorescence intensity and the best ATP production, approaching the level of the fresh group (Figure 3b, c). These findings suggest that SMF treatment might have a positive impact on cellular activity and energy metabolism. In addition, SMF‐4 is more effective in the thawing of cryopreserved OTs.

### 2.4 Analysis of DNA strand breaks and proliferation

**FIGURE 4:**
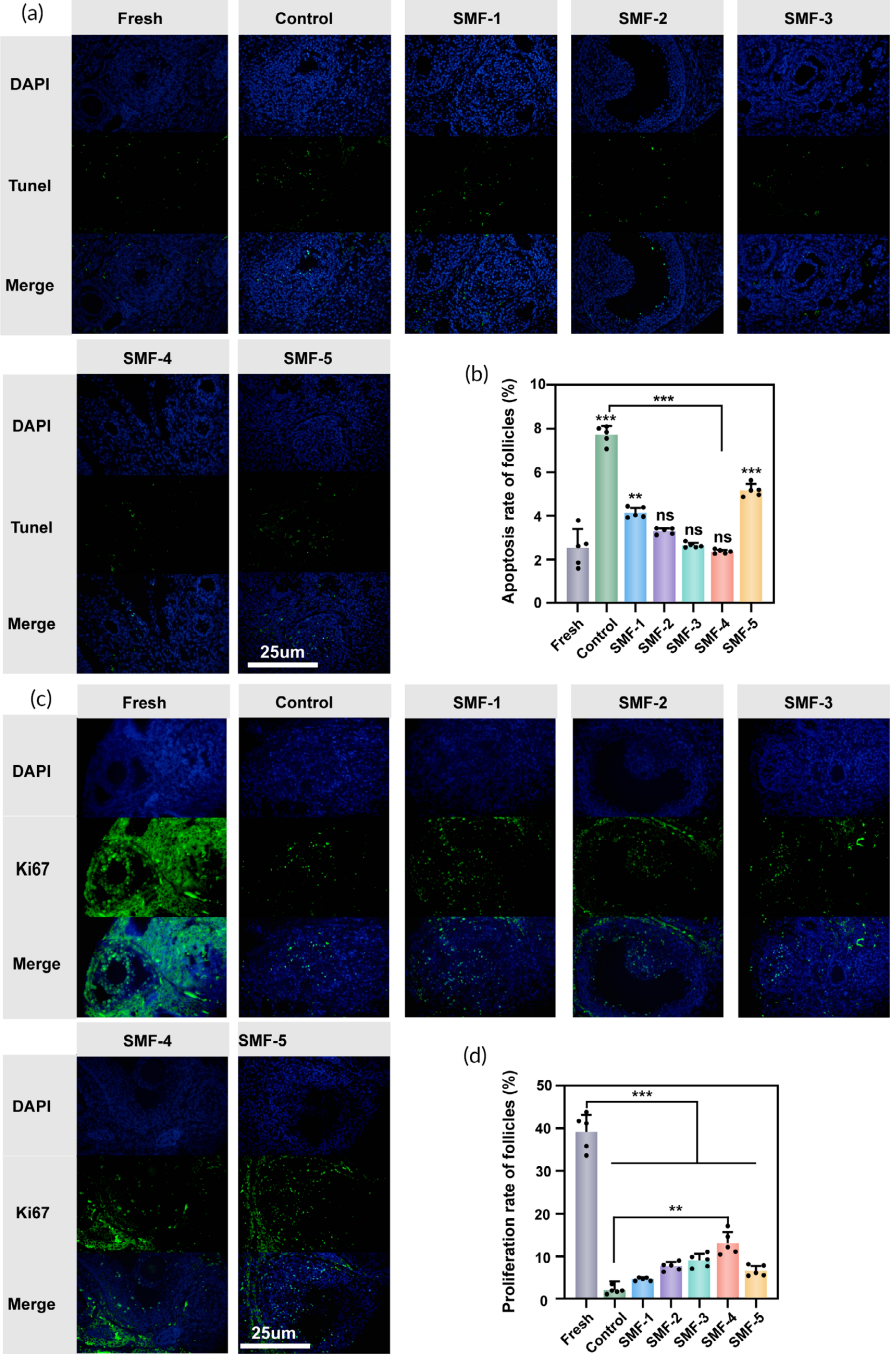
Immunohistochemical staining for Tunel and Ki67 in each group. (a) Immunofluorescence staining for Tunel, scale bar: 25 μm. (b) The corresponding quantification of Tunel. (c) Immunofluorescence staining for Ki67, scale bar: 25 μm (*n* = 5, data presented as mean ± SD). (d) The corresponding quantification of Ki67 (*n* = 5, data presented as mean ± SD).

The effect of SMFs on apoptosis and proliferation of OTs was examined through fluorescence staining. The apoptotic content in OTs was determined by the intensity of Tunel (+) signals in follicles and stroma. Representative images in Figure 4a show that the nuclei of all cells are blue, while the nuclei of apoptotic cells appear green. Fresh OTs exhibits rare green fluorescent granules and only a few apoptotic follicles. However, after cryopreservation, apoptotic cells are found in all tissues, and the green fluorescence appeared more and brighter compared to the fresh tissues. Notably, SMF‐4 shows a substantial decrease in the proportion of Tunel (+) cells, suggesting that SMFs of appropriate intensity can effectively regulate apoptosis in cryopreserved OTs (Figure 4b).

Ki67 fluorescence staining assessed the proliferation of cryopreserved OTs. Figure 4c shows the expression of Ki67. In all groups, the nuclei of all cells appear blue, while the nuclei of proliferating cells are green. The percentage of Ki67‐positive cells was quantified and analyzed (Figure 4d). In the fresh OTs, brighter fluorescent cells indicating proliferation were observed. However, cryopreservation resulted in a significant reduction in the proliferation rate of follicles compared to the fresh group (*p* < 0.001) (Figure 4b, d). Ki67 expression is significantly increased in the SMFs group compared to the control group, especially in the SMF‐4 group.

### 2.5 Analysis of transplantation

**FIGURE 5:**
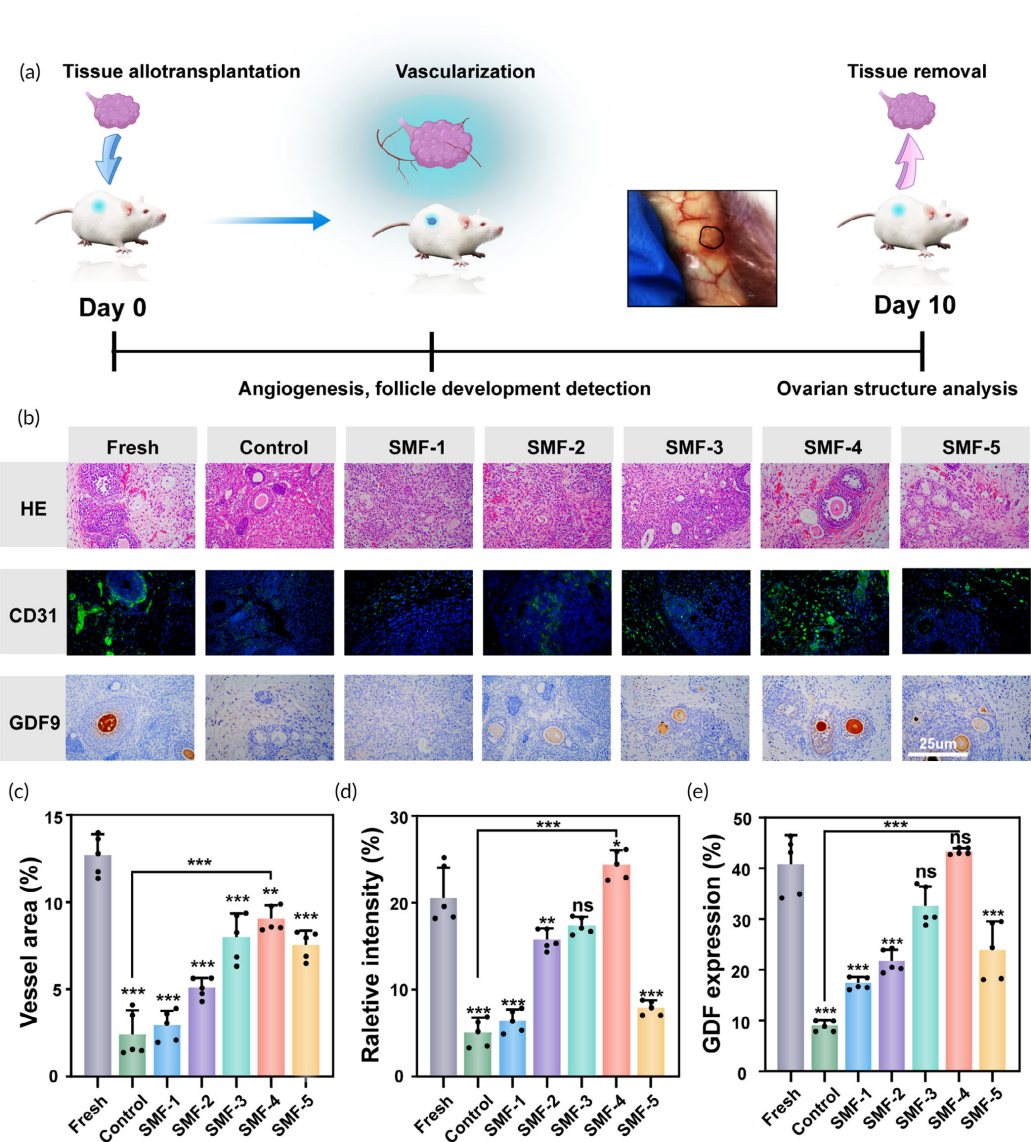
Structural and functional analysis of tissues from different groups after 10 days in vivo. (a) Timeline of OT transplantation (b) H&E staining, immunofluorescence staining for CD 31 (green), and immunohistochemistry staining for GDF 9. (c) The corresponding quantification of vessel (*n* = 5, data presented as mean ± SD). (d) The corresponding quantification of CD31 (*n* = 5, data presented as mean ± SD). (e) The corresponding quantification of GDF 9 (*n* = 5, data presented as mean ± SD).

To assess the feasibility of clinical transplantation of cryopreserved ovarian tissues (OTs), an in vivo transplant bioassay, considered the gold standard for functional evaluation, was conducted (Figure 5a). Ten days after transplantation, the OTs in the mice were located at the transplantation site, and revascularization was observed in the surrounding area (Figure S3). Specific parameters of related indicators are listed in Table S2. H&E staining (Figure 5b, c) shows that after 10 days, samples from all groups showed the generation of functionalized blood vessels. The SMF groups show better angiogenesis compared to the control group. Also, SMF‐4 exhibits a larger area of angiogenesis, similar to that observed in the fresh group. These results suggest that MF assisted‐thawing promotes tissue viability.

The quality of angiogenesis was further assessed by immunostaining for CD31 (Figure 5b). The fresh group demonstrates the highest degree of revascularization on day 10 compared to the other groups (Figure 5d). Angiogenesis increased from SMF‐1 to SMF‐3 groups and SMF‐5 but remained significantly lower than SMF‐4 group. These results indicate that SMF‐assisted thawing with an appropriate intensity can promote angiogenesis and reduce ischemia–reperfusion injury after OT transplantation in a coordinated manner.

The developmental function of the transplanted OTs further confirmed the excellent effect of the SMF. In Figure 5b, it can be observed that there was almost no trend of GDF9 expression was observed in the control group. However, the SMF groups exhibit some treatment effect. The SMF‐1 and SMF‐2 groups with lower magnetic induction intensity showed only a mild developmental promotion effect (Figure 5b). And, the GDF 9 expression rates in the SMF‐3 and SMF‐4 groups were 32.59±3.82 and 43.37±0.62, respectively (Figure 5e).

### 2.6 Active oxygen determination

**FIGURE 6:**
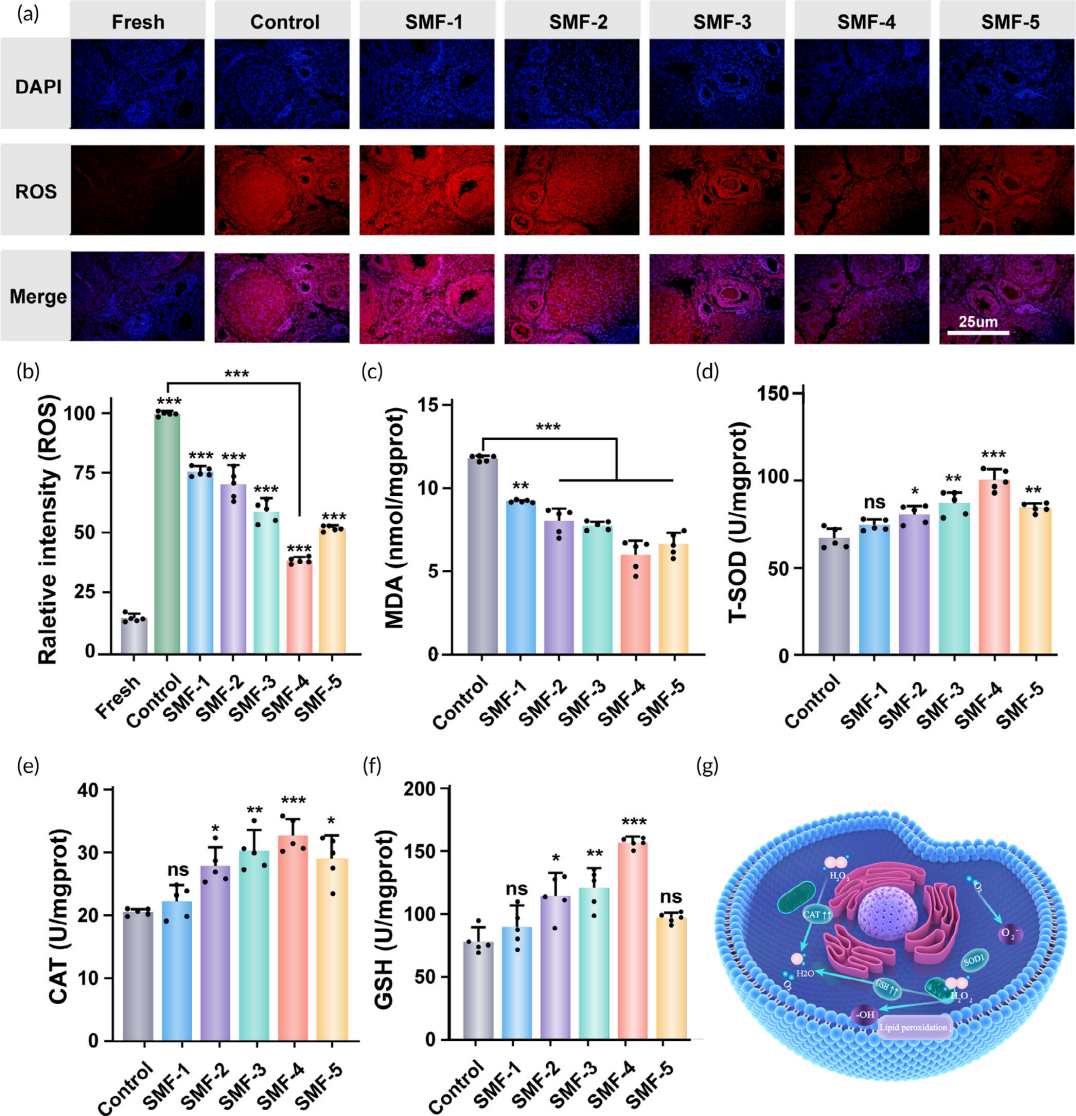
Oxidative scavenging activity of cryopreserved OTs with different thawing treatments. (a) Immunofluorescence staining for ROS (red), and nucleic (blue), scale bar: 25 μm. (b) The corresponding quantification of ROS (*n* = 5, data presented as mean ± SD). (c) MDA content (*n* = 5, data presented as mean ± SD). (d) T‐SOD content (*n* = 5, data presented as mean ± SD). (e) CAT content (*n* = 5, data presented as mean ± SD). (f) GSH content (*n* = 5, data presented as mean ± SD). (g) Schematic illustration of the ROS scavenging process with SMF.

To reveal how SMF improves the quality of cryopreserved OTs, the levels of ROS and critical substances were examined, along with specific parameters of related indicators (Table S3). Immunofluorescence analysis was performed to detect the expression levels of ROS (Figure 6a). The control group showed significantly higher ROS expression compared to the fresh group, while the SMF groups exhibited better ROS clearance, with SMF‐4 showing the most pronounced antioxidant effect (Figure 6b).

The antioxidant effect of SMF on the tissues may be attributed to its scavenging function on ROS. Analysis of critical molecules involved in ROS scavenging reveals that the SMF‐treated groups have the lowest malondialdehyde (MDA) values, indicating reduced cell membrane damage compared to the control group (Figure 6c). The percentage of T‐SOD, catalase (CAT), and glutathione (GSH) in the SMF groups are significantly higher than in the control group (Figure 6d–f). These results indicate that the presence of the SMF significantly reduced oxidative stress in the cryopreserved tissues, as schematically represented in Figure 6g. SMF‐4 appeared to be the most effective among the different treatment groups.

## 3 DISCUSSION

The ovary is a pivotal organ in the female reproductive system, and the ovarian follicle is an essential functional tissue unit in the ovaries of mammals, including humans and peromyscus.^39^ The decline in ovarian function is attributed to adverse effects of cryopreservation on tissue, including disruption to the plasma membrane and mitochondrial dysfunction. Classic CPAs are failing to dispose of ROS, while antioxidants can turn ROS into harmless materials and regulate oxidative stress.^40^ The combination of antioxidants and CPAs can improve the efficiency of cryopreservation, however, it is important to note that the misuse of antioxidants may lead to negative outcomes.^41^ In this paper, SMF were applied during thawing to prevent the harmful effects of the cryopreservation process on mouse ovaries. And, our results suggest that the presence of SMF not only increased the morphological health of follicles after cryopreservation and maintained the percentage of collagen fibers, but also enhanced the viability of the transplanted tissue. Furthermore, our findings indicate that SMF has the potential to effectively protect ovarian tissue against oxidative damage.

During cyropreservation, mitochondrial dysfunction can lead to an imbalance in oxidative status, resulting in changes in tissue structure and morphology, ultimately leading to follicular loss. Our data indicate that follicle morphology and ovarian matrix collagen fiber content both decrease after cryopreservation compared to fresh tissue, consistent with previous studies.^21^, ^42^ The thawing protocol used in this study had beneficial effects on follicular morphology. However, the findings of the current study do not support the previous research. Researchers compared three different magnetic field treatments (equilibration stage,  freezing stage, and thawing stage) for cryopreservation of ovarian tissue.^38^ In contrast to this paper, they only used a single gradient magnetic field exposure (1 mT) without comparing it to multi‐gradient magnetic field treatment.^38^ They concluded that MF treatment during the thawing period had a negative impact on follicular morphology compared to the fresh control group.^38^ The decrease in the proportion of normal follicles may be primarily attributed to the differences in the thawing solution, which lacked concentration gradient changes compared to the present study. It is generally known that the loss rate for intact follicles during cryopreservation is significant.^43^ SMF may provide better protection for ovarian stromal integrity, which may help maintain more morphologically normal follicles. This could be attributed to the ability of MF to maintain the oxidative balance of fibroblasts within the tissue, thereby helping to preserve collagen fiber content and maintain a higher number of follicles with normal morphology.^36^, ^37^ The ECM structure allows for a specialized microenvironment to regulate cellular activity and development, including the regulation of cell proliferation and survival, and morphology.^44^, ^45^, ^46^, ^47^ Surprisingly, it has been observed that MF can increase the mechanical properties of tissues. Several factors may contribute to this observation. Firstly, magnetic field treatment may lead to a decrease in medullary porosity within the tissues, resulting in a more compact and intact tissue structure. Additionally, magnetic field exposure has the potential to preserve the collagen fiber content within the tissues, which can contribute to improved mechanical properties. The results obtained suggest that appropriate magnetic induction intensity can help protect the function of preserved ovarian tissue and maintain its follicular development potential.

In the normal process of follicular development, GCs play a crucial role in supporting oocytes by providing essential nutrients, signaling molecules, and metabolic products during oocyte development and maturation.^37^, ^48^, ^49^ Studies have demonstrated the significant protective effects of granulosa cells on oocytes, particularly immature oocytes.^36^ An alternative explanation for this result is that the gradual penetration of CPAs by GCs, preventing damage to oocytes caused by sudden osmotic pressure changes during the thawing process. As a result, oocyte survival and fertilization rates are significantly improved. Research has shown that vitrification cryopreservation of oocytes with intact GCs reduces damage to the cytoskeleton, improves oocyte recovery, and enhances the developmental outcomes of GV‐stage oocytes.^49^ Elevated levels of apoptosis within GCs can lead to follicle death, ultimately affecting reproductive efficiency. A notable finding from our analysis is that oocytes and GCs treated with a SMF exhibited improved conditions, with a reduced apoptosis rate. These results corroborate the findings of a great deal of the previous work.^50^ They discovered that a SMF improved mitochondrial membrane potential activity and the ultrastructure of cryopreserved pre‐antral follicles in mice, resulting in enhanced embryo cleavage rates. A possible explanation for this might be that SMFs modulate DNA damage and/or damage repair, possibly through a mechanism that affects mitochondria.^50^, ^51^ The state of mitochondrial activity and ATP content in the tissue indicate that the magnetic field has the potential to enhance energy production within the cells. This increase in energy production can lead to heightened cellular activity and improved cell survival rates.

Indeed, in early studies, it has been suggested that SMFs can reduce apoptotic cell death caused by various factors in different human cell types. The underlying mechanism behind this result is difficult to explain, but it might be related to the regulation of the oxidative state of internal processes within tissues.^52^ To date, SMF has been found to be effective in regulating the oxidation–reduction homeostasis of cells. In accordance with our observations, Wei et al. found that MF therapy significantly reduced ROS accumulation and MDA production in the radiation‐injured model, while increasing the activity of antioxidant enzymes.^53^, ^54^ As mentioned in the literature review, the inhibitory effect of SMFs on Fe^2+^‐induced ROS generation is limited to a field strength “window” of 2 to 4 mT.^55^ A strong relationship between ROS and the efficacy of cryopreservation has been reported.^56^ Additionally, ROS can induce apoptosis or dysfunction and, ultimately leading to a reduction in oocyte survival and developmental ability.^19^ The most striking finding from our analysis is the correlation between ROS content and the degree of tissue damage. The increase in ROS content within tissues may be attributed to intrinsic factors during the cryopreservation, such as the toxicity of CPAs, rapid temperature decreases, osmotic pressure changes, as well as non‐physiological conditions or surgical procedures in the extracellular environment.

The antioxidant system consists mainly of a series of enzymatic substances. SOD is the primary line of defense to counteract ROS damage by reducing excessive concentrations of ROS and safeguarding cells from injuries.^57^ CAT is the second line of defense against ROS damage.^57^ GSH is an antioxidant sub‐state in the body, and its deprivation is related to increased oxidative damage.^58^ Moreover, the disruption of the prior studies that have noted the importance of glutathione that can alleviate the detrimental effects of vitrification.^58^, ^59^ ROS/GSH balance causes harmful oxidation and chemical changes of biomacromolecules, leading to cell‐cycle arrest, suppression of proliferation, and even cell death.^58^ This is consistent with our results. The treatment with SMFs results in increased levels of the common antioxidant factors T‐SOD, CAT, and GSH, thereby significantly reducing ROS levels. These agree with other research work, thus support the idea that SMF has a free radical scavenging effect.^36^, ^50^, ^60^ Research has shown that oxidative stress induced by cryopreservation leads to extensive damage to cell membranes, resulting in an increase in MDA levels.^25^, ^61^, ^62^, ^63^ However, when applying SMF to reduce ROS levels, it can help downregulate the expression of MDA and protect the integrity of cell membranes. In the present study, we confirmed that cryopreserved OTs assisted by SMFs for thawing exhibited potent antioxidant properties. Since this, it can be hypothesized that the ROS scavenging abilities of SMF can improve the quality of tissues.

OT transplantation is a common method to maintain fertility in young patients undergoing chemotherapy/radiation.^29^ However, ischemia–reperfusion injury is one of the main limitations of this technique and can result in follicular depletion within the transplanted fallopian tube.^64^ To reduce this damage and protect the follicular pool, it has been proposed that accelerated angiogenesis early after transplantation could be achieved by reducing the duration of ischemia.^65^ Previous studies have shown that the dorsal muscle serves as a graft site known to accelerate angiogenesis.^66^ Here, OT was transplanted to the dorsal side of allogeneic mice. A lower oxidative stress environment was hypothesized to promote angiogenesis in the grafts.^67^ Results from treating samples with SMFs via transplantation were favorable, as evidenced by the upregulation of angiogenesis. The medulla is the central part of the ovarian parenchyma and contains blood vessels that supply nutrients. The magnetic field‐treated group showed a lower medullary porosity, indicating a lower degree of vascular damage. This reduced porosity may contribute to post‐transplantation vascular regeneration and decrease the rate of follicular loss. Another possible explanation for this is that the generation of free radicals and inflammation before transplantation leads to apoptosis of oocytes in the transplanted ovaries.^29^ During folliculogenesis, oocytes can regulate follicle growth through paracrine factors, particularly GDF 9, which affects the proliferation of granulosa cells.^68^ As evidenced by our results, the increased GDF 9 expression in the treated group may be due to the effects of SMFs on oocyte maturation and follicle development, as previously suggested by Baniasadi et al.^38^ Modulating of the microenvironment within the tissue may have resulted in better proliferation, vascularization, and histomorphology.

Our research findings indicate that the effects of applying SMF during ovarian tissue re‐warming may reach optimal outcomes at specific intensity. Improvement in ovarian tissue quality parameters was observed at a SMF intensity of 40 GS, but similar effects were not observed at higher intensity. This could be attributed to the counteractive effects at higher magnetic induction intensity, where higher intensity magnetic fields may not provide additional benefits. It is possible that higher intensity could have detrimental effects on tissue cells by altering the movement of charged substances within the tissue or changing the state of cell membranes. Future research should further investigate these mechanisms to understand the potential impact of SMF on ovarian tissue activity.

## 4 CONCLUSION

This study developed a novel technique, SMF‐assisted thawing, which improves the antioxidant damage capacity for optimizing whole OT cryopreservation and its transplantation. It is found that SMF‐assisted thawing preserves follicle morphology in OT by maintaining collagen fiber content within the stroma. Moreover, there are enough intact mitochondria after cryopreservation with SMF through the analysis of the ultrastructure of granulosa cells. And thus, it can maintain the complete cellular respiratory chain, thereby reducing the production of ROS in vivo. Furthermore, the results indicate that the SMF can increase the antioxidant capacity, reduce the production of ROS in OT, subsequently reduce the tissue apoptosis, and promote angiogenesis in post‐transplant tissues. In conclusion, with SMF applied, the antioxidant substances in the OTs can be induced to increase and then the tissue can resist the oxidative stress injury. This finding provides new insight into the cryopreservation of female fertility. We are confident that it will have good application prospects in the field of assisted reproduction and cryopreservation of other tissues and organs.

## 5 MATERIALS AND METHODS

### 5.1 Ovarian tissue preparation

KM mice aged 6–8 weeks were sacrificed in all experiments, weighing approximately 30 g each. Both ovaries were removed from the mice and cleared from fat tissue. The procedure was performed between 9 and 10 a.m. The removed ovaries were immediately transferred to ethanol (70%), washed for about 10 s, and then transferred to 0.9% saline and rinsed twice, which was supplemented with penicillin (100 μg mL^−1^) and streptomycin (100 μg mL^−1^). After rinsing, the ovaries were immersed in a 50 mL centrifuge tube containing DMEM medium and sent to the laboratory within 20 min. DMEM medium was supplemented with penicillin (100 μg mL^−1^) and streptomycin (100 μg mL^−1^). In this case, some samples were directly fixed in 4% paraformaldehyde for subsequent experiments. These samples belong to the fresh group. All chemical reagents used in this study were purchased from Hefei Yuanen Biotechnology Co., Ltd. (Hefei, China) unless otherwise stated. The study was approved by the Experimental Animal Ethics Committee of Anhui Medical University (No. LLSC20220725).

### 5.2 Static magnetic field generator

The experimental setup of the SMF generator consists of a pair of Helmholtz coils and an ADC regulated power supply, which can generate the stable magnetic field required during the experiment (Figures S4 and S5). The specific parameters of the coils and their use are described in reference.^69^ Based on the parameters of the coils, the magnetic field distribution in the two cross sections where the samples were located inside the coils was simulated using COMSOL software (v. 6.1, COMSOLAB, Stockholm, Sweden). To provide more information on how the simulations were performed, we utilized the AC/DC module to conduct a three‐dimensional simulation. However, we acknowledged that we omitted some non‐magnetic materials such as the acrylic frame and polyethylene support components. Regarding the governing equations of the system, we focused on the key area of the internal coil during the simulation of the static magnetic field.

The governing equations used in the system were:

▽xH=J

B=▽xA

J=σE

We recognized that the magnetic field intensity changes significantly in this area, requiring higher precision in the simulation. Therefore, we improved the accuracy of our simulation results by locally refining the mesh within the internal coil to calculate the magnetic field distribution and intensity more precisely. To demonstrate that the OT sample inside the cryotube was subjected to uniform magnetic field, the magnetic induction intensity where the tissue located was measured. We fixed the real‐time inspection equipment—Gaussmeter at the position. And the magnetic induction intensity at the location of the sample was measured every 5 min, to assess the stability of the magnetic field during experiment. In order to avoid the effect of the magneto‐generated heat phenomenon on the tissue, we took measures to monitor the heat production using thermocouples placed inside the coil during the experiment.

### 5.3 Freezing of intact ovaries

The cryopreservation protocol of OT in this study was performed as described by Jasemi with minor modifications, as illustrated in Scheme 1a.^70^ Briefly, each ovary is equilibrated in two steps: immersion in a bath containing different concentrations of CPA. Tissues were exposed to cryopreservation solution 1 (10% DMSO and 0.5 m sucrose) for 5 min, then transferred to solution 2 (20% DMSO and 0.5 m sucrose) for 5 min, followed by removal of excess CPAs with medical gauze.^71^ The ovaries were placed on gauze to carry the least amount of medium in each transfer during the cryopreservation procedures. The ovaries were immersed directly into liquid nitrogen (LN_2_) to achieve rapid freezing. Finally, the ovaries were transferred to a 1.5 mL cryotube and stored in LN_2_ for at least 2 weeks.

### 5.4 Thawing of intact ovaries

During thawing, the ovarian tissue was directly transferred from the cryotube into a pre‐warmed thawing solution. The thawing solution has been preheated in a water bath at 37°C. The ovaries were bathed in three steps, for 5 min in each, at room temperature (RT), with stepwise dilutions of 0.5, 0.25, and 0 m sucrose in DMEM medium, to avoid osmotic injuries.

In this case, the ovaries were allocated as follows: (1) control group, and (2) SMF assisted‐thawing group. During the entire thawing process (**Scheme** **1**b), the same thawing solution was used, but different Static Magnetic Field (SMF) strengths of 10 Gs, 20 Gs, 30 Gs, 40 Gs, and 50 Gs were applied. After thawing, some samples of each group were fixed with 4% paraformaldehyde for subsequent experiments.

### 5.5 Morphological analysis by hematoxylin and eosin

Mouse ovaries from different thawing and fresh control groups were fixed in 4% paraformaldehyde for 24 h.^72^, ^73^ The tissues were embedded in paraffin for serial sectioning at a thickness of 5 μm. In addition, hematoxylin and eosin (HE) staining were performed using standard procedures. After slicing, the automatic digital slide scanner scans the slices. Sections were observed under light microscopy (×4 and ×200) (Nikon, Tokyo, Japan). In addition, the images were analyzed using ImageJ software to determine the porosity of the OT medulla.

### 5.6 Extracellular matrix analysis by Sirius red

To assess the distribution of collagen fibers in the ECM, ovarian cortical tissue was stained with Picrosirius Red (Abcam kit). Briefly, 5.0 μm ovarian sections were soaked three times in xylene and similarly washed three times using a gradient of reduced ethanol (100%–95%–80%). After removing excess ethanol with a stream of water until the sections were clean and clear, they were incubated for 1 h at room temperature using Sirius Red solution (0.1%). The final conventional dehydration was transparent and evaluated under a light microscope (Nikon, Eclipse, TS 100, Japan) at 400× magnification. For each group, the percentage of area in five different regions with collagen fibers was processed by a DS‐cooled camera head DS‐Ri1 attached to a microscope (Nikon, Eclipse, TS 100. Japan).

### 5.7 Mechanical properties testing

The samples are placed at the center of the platform of the tensile testing machine (Figure S2d). The testing distance of the machine is adjusted so that the upper platform of the machine slowly descends. The movement is stopped when the surface of the upper platform is about to come into contact with the sample (approximately 1–3 mm). The testing software on the computer is opened, and both the force and displacement output signals are zeroed. The preset strain rate is set, and the axial strain of the sample is increased at a rate of 1 mm/min. The maximum loading strain is set at 30% for compression testing. Throughout the entire experiment, the stress–strain data are automatically collected and recorded using the ZQ990‐B software on the computer. The compression testing samples are used for a single experiment only and cannot be reused after the experiment. All uniaxial compression experiments are conducted at room temperature. The compression testing should be completed as quickly as possible to prevent prolonged exposure of the samples to air, which may affect the experimental results.

### 5.8 Ultrastructural evaluation by transmission electron microscopy

After the whole ovary was warmed, tissues were taken for ultrastructural evaluation. Samples were cut into small pieces of about 1 mm^3^, fixed in 2.5% glutaraldehyde fixative for the tissue electron microscope, and then placed in a refrigerator at 4°C for 3 days. The fixed tissue blocks were treated with 0.1 m phosphate buffer pH 7.0 to remove the fixative, then fixed with 1% osmic acid solution for 2 h. Tissue pieces after PBS rinsing were dehydrated with a gradient concentration of ethanol solution and then treated with pure acetone instead of solvent. The treated sample is then embedded, sliced, and stained. After inserting the cubes into Epon812 resin blocks, blocks were incubated in 65°C for 2 days. Thereafter, blocks were cut into ultrathin sections (70–100 nm thickness) using ultramicrotomes (Leica EM UC7). For obtaining ultrastructure photos, ultrathin sections were finally moved to 200‐mesh uncoated grids then stained by lead citrate. Finally, the transmission electron TEM image was obtained using a Thermo scientific Talos L120C G2 transmission electron microscope.

### 5.9 ATP analysis

In order to evaluate the function of mitochondria, the expression of ATP in tissues was observed using immunofluorescence technique after thawing. The tissue sections were hyalinized and then subjected to antigenic high‐pressure repair. The sections were then closed with goat serum. Rabbit anti‐ATP citrate lyase antibodies (1:200, Abcam, China) were incubated at 37°C for 1 h, followed by goat anti‐rabbit antibodies (1:300, Abcam, China) at 37°C for 30 min under light. Sections were then thoroughly cleaned with PBS before the nuclei were stained with DAPI (Beyotime, China) solution. The percentage of ATP expression was calculated using ImageJ software and a light microscope to examine the sections.

### 5.10 Analysis of DNA strand breaks by TUNEL

According to the manufacturer's instructions, TUNEL staining was performed with a Tunel kit (Beyotime, China). The glass slides were soaked in xylene and then hydrated with gradient ethanol. After washing with tap water, the tissues were treated with proteinase K solution at 37°C. After rinsing with PBS, the tissues were added with 50 μL of prepared tunel assay solution, and then rinsing was continued with PBS. After restaining the cell nuclei with DAPI (Beyotime, China), the sections were observed with a microscope and photographed (BA410E, Motic, China).

### 5.11 Proliferation analysis

The sections were sectioned and treated with xylene and a reduced ethanol gradient, followed by slow rinsing with tap water until the sections were clean and clear. After antigen repair with high pressure, the tissues were blocked with goat serum for 30 min. After incubation with the rabbit Ki67 antibody (Affinity, China), the blocked tissues were rinsed three times with PBS and then incubated with the goat anti‐rabbit antibodies (1:300, Abcam, China). Finally, the sections were subjected to DAPI (Beyotime, China) re‐staining, and the sections were scanned by an automated digital slide scanner. Proliferation was analyzed by calculating the percentage of positive follicles in the five corresponding microscopic fields.

### 5.12 ROS analysis

First, Dihydroethidium (Beyotime, China) needs to be dissolved in DMSO (Biosharp, China) and prepared into an appropriate stock solution of 5 mg/mL. Then, for probe labeling, slice sections are taken out and routinely dewaxed in water. Next, immunohistochemical pen strokes are performed, and tissue autofluorescence quenchers are added. Following this, 100 μL of staining solution (DHE‐1:1000 dilution) is added, and the sample is incubated in a light‐avoiding incubator at 37°C for 30 min. Afterwards, the staining solution needs to be removed, and the sample is washed three times with PBS and stained with DAPI (Beyotime, China) for 5 min. Then, the slice is washed with PBS 2–3 times, and an anti‐fluorescence quencher (Beyotime, China) is added to seal the sample. Finally, the sample is examined using a fluorescent microscope.

### 5.13 Active oxygen determination

OTs were prepared as 10% tissue homogenates by normal saline and centrifuged at 2500 rpm at 4°C for 10 min. The supernatant was collected for further analysis. The concentrations of T‐SOD, GSH, CAT, and MDA in OTs were measured according to the manufacturer's instructions using Antioxidant kits (Nanjing Jiancheng Bioengineering Institute). Absorbance at the corresponding wavelength was determined using a microplate reader.

### 5.14 Analysis of transplantation

OT transplantation was performed according to an approved protocol. The recipient mice did not undergo ovariectomy (removal of ovaries) prior to the transplantation. As illustrated in Scheme 1c, the backs of the mice were shaved and sterilized after being given anesthesia. Small incisions were made in the skin and peritoneum on both sides of the body in the last third of the spine, and thawed ovaries were immediately transplanted into the back, with one tissue being implanted through each incision. 10 days after transplantation, the mice were anesthetized to death by overdose. The grafted tissue was removed, fixed at room temperature for 2 days using 4% paraformaldehyde, and paraffin‐embedded. And we stained with hematoxylin and eosin, CD31 immunofluorescence, and GDF9 immunohistochemistry according to standard protocols. Images were obtained using an inverted microscopy and quantitatively analyzed using ImageJ software.

### 5.15 Statistical analysis

All analyses were performed using OriginPro. The data are presented as the mean ± SD, and the results presented were representative data sets. Where appropriate, a one‐way analysis of variance (ANOVA) and the Student's *t*‐test were performed to assess statistical significance. For all tests, *p* values are as follows: ns = non‐significant (*p* > 0.05); * = statistically significant (*p* < 0.05); ** = statistically significant (*p* < 0.01); *** = statistically significant (*p* < 0.001). Two‐sided statistical tests were performed in all statistical analyses.

## AUTHOR CONTRIBUTIONS

**Liyuan Zhang:** Data curation (lead); formal analysis (equal); investigation (lead); writing – original draft (lead); writing – review and editing (lead). **Mengqiao Chi:** Data curation (equal); formal analysis (equal); investigation (supporting). **Yue Cheng:** Conceptualization (equal); formal analysis (equal); project administration (equal). **Zhongrong Chen:** Conceptualization (lead); formal analysis (lead); funding acquisition (equal); project administration (lead); supervision (equal); writing – original draft (equal); writing – review and editing (lead). **Yunxia Cao:** Formal analysis (equal); writing – review and editing (equal). **Gang Zhao:** Conceptualization (lead); data curation (lead); formal analysis (equal); funding acquisition (lead); supervision (lead); writing – review and editing (lead).

## CONFLICT OF INTEREST STATEMENT

The authors declare that they have no known competing financial interests or personal relationships that could have appeared to influence the work reported in this paper.

### PEER REVIEW

The peer review history for this article is available at https://www.webofscience.com/api/gateway/wos/peer-review/10.1002/btm2.10613.

## Supporting information


**DATA S1.** Supporting Information

## Data Availability

The data that support the findings of this study are available from the corresponding author upon reasonable request.
