# Supplementary material for: Static magnetic field assisted thawing improves cryopreservation of mouse whole ovaries
Source: Bioeng Transl Med. 2023 Oct 18;9(1):e10613. doi: 10.1002/btm2.10613 (PMC10771557; doi:10.1002/btm2.10613)
Supplement: Supplementary file 1 — DATA S1. Supporting Information [file BTM2-9-e10613-s001.docx]

**Supplementary Material**

**Static magnetic field assisted thawing improves cryopreservation of mouse whole ovaries**

*Liyuan Zhang ^1^, Mengqiao Chi ^1^, Yue Cheng ^2^, Zhongrong Chen^2 *^, Yunxia Cao^3,4^，Gang Zhao^2,5 *^*

*^1^* School of Basic Medicine, Anhui Medical University, Hefei, China

*^2^* Research and Engineering Center of Biomedical Materials, School of Biomedical Engineering, Anhui Medical University, Hefei, China

*^3^* Reproductive Medicine Center, Department of Obstetrics and Gynecology, the First Affiliated Hospital of Anhui Medical University, Hefei, China

*^4^* NHC Key Laboratory of study on abnormal gametes and reproductive tract (Anhui Medical University), Hefei, China

*^5^* Department of Electronic Engineering and Information Science, University of Science and Technology of China, Hefei, China

* Corresponding authors.

E-mail addresses: zhaog@ustc.edu.cn; [zhrchen@ahmu.edu.cn](mailto:zhrchen@ahmu.edu.cn)

**Supplementary Figures**





**Figure S1**

Heat generation inside the magnetic field generator as a function of time.


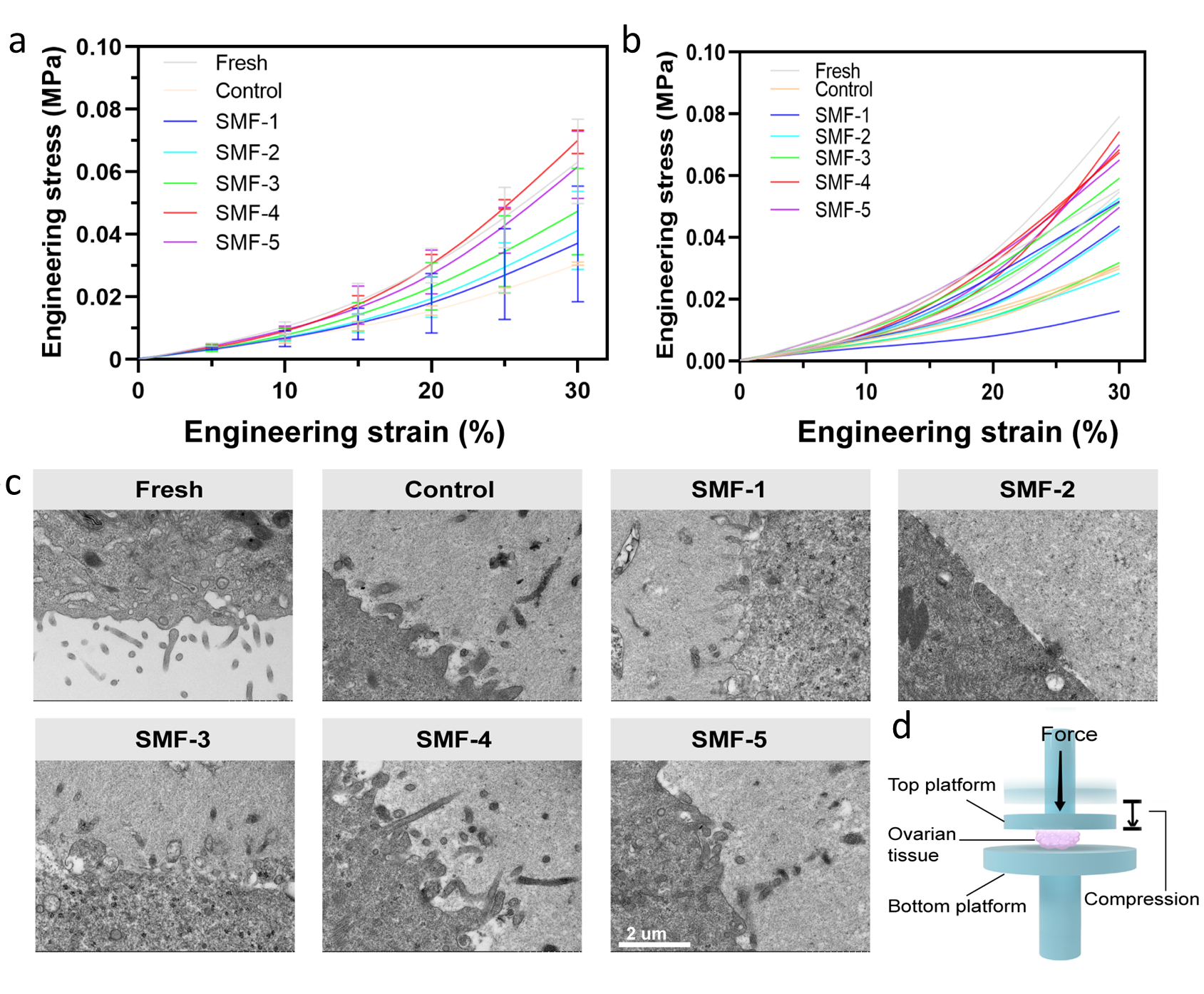


**Figure S2**

a&b. Compression curve of ovarian tissue (n=3). c.Transmission electron microscopy of mouse oocyte in cryopreserved and cryopreserved-warmed groups at the presence and absence of static magnetic field. d. Compression experimental schematic diagram.


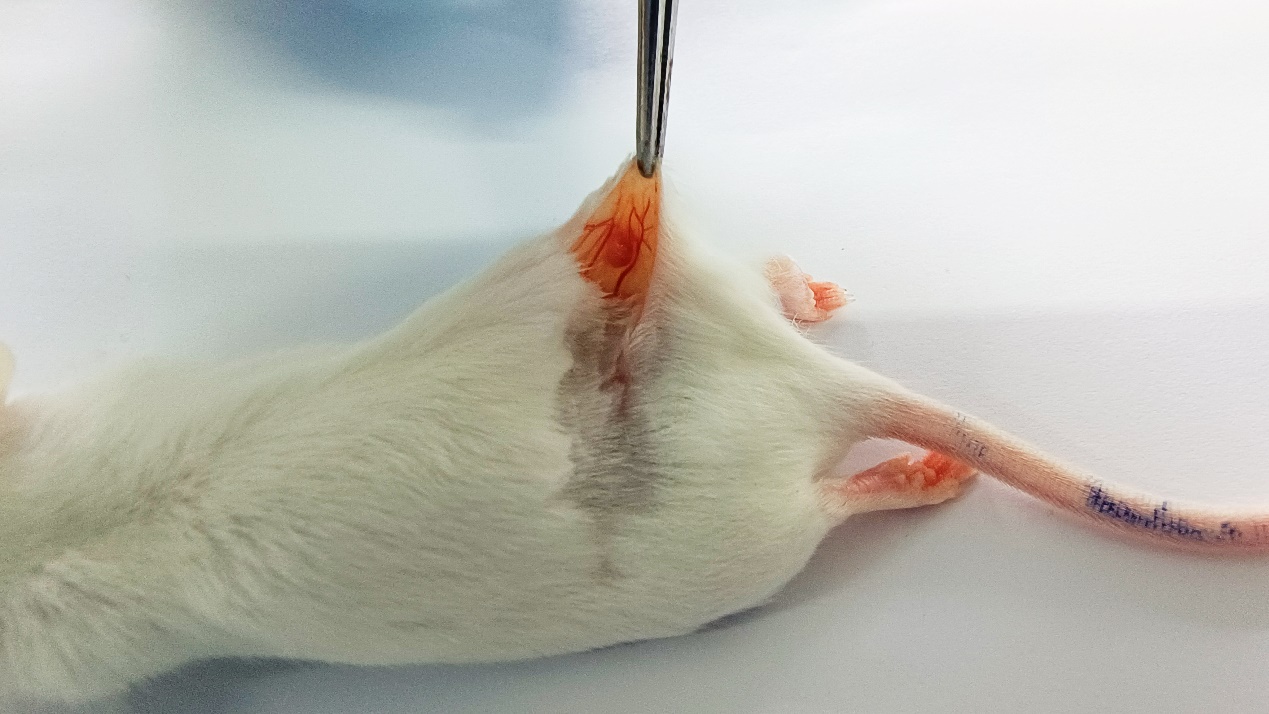


**Figure S3**

The location of ovarian tissue and perivascular reconstruction in mice after transplantation.

**
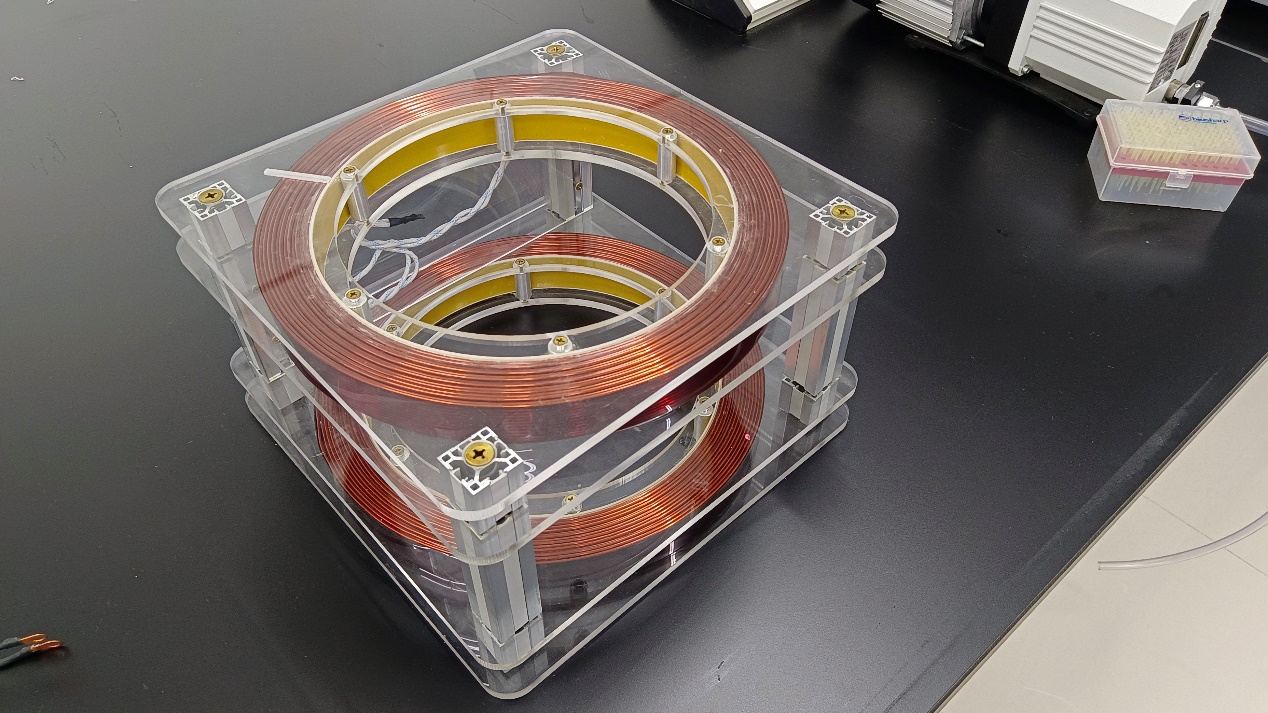
**

**Figure S4**

Physical drawing of magnetic field generating device.

**
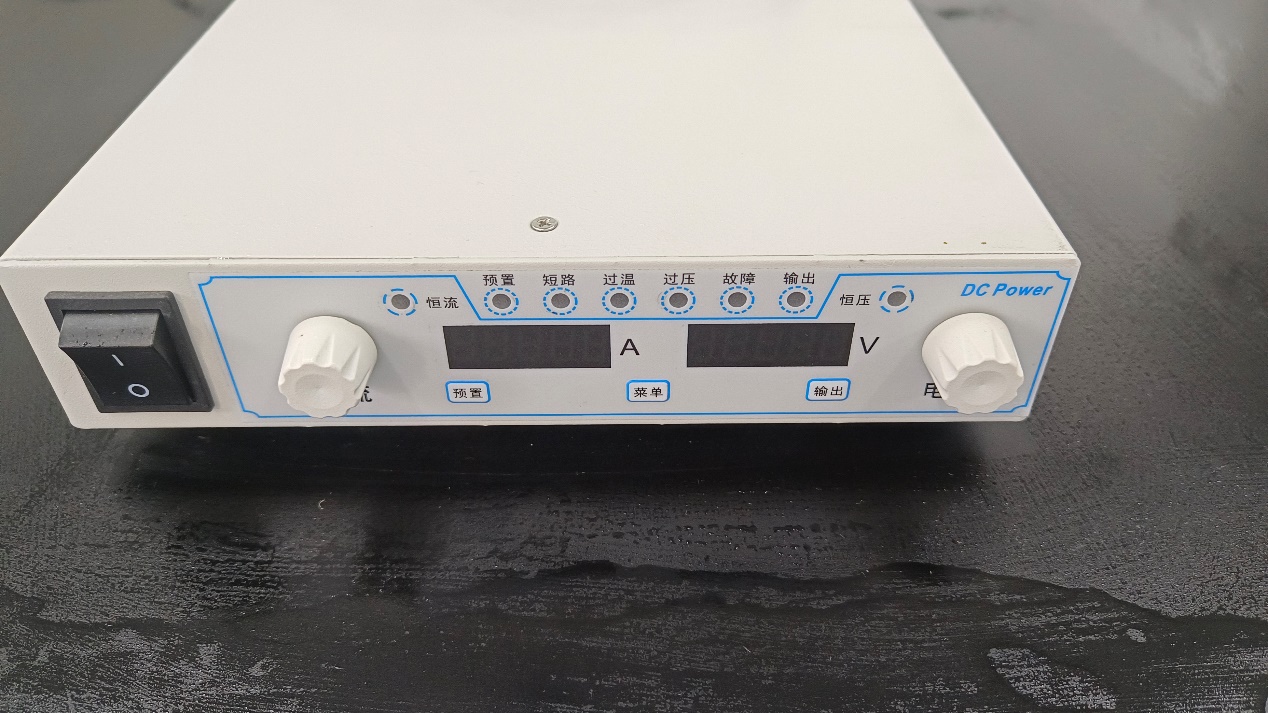
**

**Figure S5**

Physical drawing of DC regulated power supply.

**Supplementary Tables**

**Table S1** Morphological and functional parameters of ovarian tissue were measured in different groups (n=5/group).

|  | Medullary pores (%) | Collagen fiber content (%) | OT apoptosis (%) | OT proliferation (%) | Quantification of ATP |
| --- | --- | --- | --- | --- | --- |
| Control | 14.45±2.75 | 10.13±0.29 | 7.79±0.40 | 2.07±1.21 | 25.78±4.65 |
| 10 Gs | 10.87±1.05 | 22.98±6.09 | 4.15±0.22 | 4.64±0.39 | 35.99±9.86 |
| 20 Gs | 9.55±0.72 | 40.86±4.28 | 3.31±0.12 | 7.64±0.39 | 59.46±18.49 |
| 30 Gs | 8.70±1.61 | 48.46±1.48 | 2.61±0.12 | 8.96±1.62 | 75.59±22.05 |
| 40 Gs | 2.79±0.47 | 55.46±3.61 | 2.33±0.08 | 13.09±2.59 | 106.19±23.27 |
| 50 Gs | 8.39±2.36 | 30.93±6.07 | 5.09±0.30 | 6.63±1.14 | 95.29±10.28 |

**Table S2** Structural and functional analysis of tissues from different groups after 10 d
in vivo (n=5/group).

|  | Microvascular area (%) | Quantification of CD 31(%) | Quantification of GDF 9 (%) |
| --- | --- | --- | --- |
| Control | 2.43±1.35 | 5.14±1.61 | 9.02±1.06 |
| 10 Gs | 2.96±0.80 | 6.35±1.27 | 17.37±1.19 |
| 20 Gs | 5.09±0.55 | 15.75±1.22 | 21.72±2.27 |
| 30 Gs | 7.98±1.36 | 17.48±0.97 | 32.59±3.82 |
| 40 Gs | 9.04±0.77 | 24.53±1.67 | 43.37±0.62 |
| 50 Gs | 7.54±0.82 | 7.86±0.85 | 23.88±5.68 |

**Table S3** Determination of oxidation-related parameters in different groups (n=5/group).

|  | Quantification of ROS | SOD (U/mgprot) | CAT  (U/mgprot) | GSH (U/mgprot) | MDA (nmol/mgprot) | |
| --- | --- | --- | --- | --- | --- | --- |
| Control | 100±1.18 | 67.43±5.35 | 20.54±0.52 | 78.17±11.67 | | 11.79±0.15 |
| 10 Gs | 75.91±2.58 | 75.07±3.06 | 22.22±2.63 | 89.77±17.52 | | 9.20±0.07 |
| 20 Gs | 70.75+8.19 | 81.12±4.65 | 27.83±2.98 | 114.4±18.71 | | 8.05±0.73 |
| 30 Gs | 59.20±6.01 | 87.33±6.05 | 30.18±3.32 | 121.0±15.94 | | 7.75±0.23 |
| 40 Gs | 39.21±1.71 | 100.7±6.33 | 32.62±2.64 | 157.3±4.60 | | 5.99±0.86 |
| 50 Gs | 52.60±1.46 | 84.66±2.66 | 28.96±3.73 | 97.40±4.01 | | 6.65±0.67 |
